# Optimization of Bone Health in Children before and after Renal Transplantation: Current Perspectives and Future Directions

**DOI:** 10.3389/fped.2014.00013

**Published:** 2014-02-24

**Authors:** Kristen Sgambat, Asha Moudgil

**Affiliations:** ^1^Children National Medical Center, Washington, DC, USA

**Keywords:** chronic kidney disease, corticosteroids, vitamin D, FGF-23, hypophosphatemia, DXA, pQCT, microbiome

## Abstract

The accrual of healthy bone during the critical period of childhood and adolescence sets the stage for lifelong skeletal health. However, in children with chronic kidney disease (CKD), disturbances in mineral metabolism and endocrine homeostasis begin early on, leading to alterations in bone turnover, mineralization, and volume, and impairing growth. Risk factors for CKD–mineral and bone disorder (CKD–MBD) include nutritional vitamin D deficiency, secondary hyperparathyroidism, increased fibroblast growth factor 23 (FGF-23), altered growth hormone and insulin-like growth factor-1 axis, delayed puberty, malnutrition, and metabolic acidosis. After kidney transplantation, nutritional vitamin D deficiency, persistent hyperparathyroidism, tertiary FGF-23 excess, hypophosphatemia, hypomagnesemia, immunosuppressive therapy, and alteration of sex hormones continue to impair bone health and growth. As function of the renal allograft declines over time, CKD–MBD associated changes are reactivated, further impairing bone health. Strategies to optimize bone health post-transplant include healthy diet, weight-bearing exercise, correction of vitamin D deficiency and acidosis, electrolyte abnormalities, steroid avoidance, and consideration of recombinant human growth hormone therapy. Other drug therapies have been used in adult transplant recipients, but there is insufficient evidence for use in the pediatric population at the present time. Future therapies to be explored include anti-FGF-23 antibodies, FGF-23 receptor blockers, and treatments targeting the colonic microbiota by reduction of generation of bacterial toxins and adsorption of toxic end products that affect bone mineralization.

## Introduction

Bone mineralization during childhood and adolescence establishes the foundation for bone health throughout life, as 90% of bone mass accrual occurs during the first 20 years. (1). Healthy bone development requires intricately balanced endocrine and mineral metabolism. In children with chronic kidney disease (CKD), this balance is disturbed, as a constellation of CKD-related factors converge to exert negative effects on bone mineralization, commonly resulting in CKD-Mineral and Bone Disorder (CKD-MBD) prior to transplant. Renal transplantation may correct many of the underlying CKD risk factors, but introduces new risk factors as well. Consequently, pediatric renal transplant recipients remain at increased risk for low bone mineral density (BMD) (2, 3). As function of the renal allograft declines over time, CKD-specific risk factors resurface, further impairing bone health. Consequences of bone disease in children with CKD may include fractures, bone pain, growth impairment, skeletal deformities, soft-tissue calcifications, increased risk of cardiovascular disease, and decreased quality of life and life expectancy. Thus, optimization of bone health is critical in pediatric patients facing a lifetime with CKD. Pre- and post-transplant risk factors, current evaluation and management strategies, as well as emerging future therapies will be reviewed.

## Bone Development and Evaluation

In growing children, bone formation is characterized by two different processes: modeling and remodeling. Bone modeling is unique to growing children; this process promotes new bone formation at sites different from those of bone resorption, resulting in increase in bone mass, changes in bone dimensions, and longitudinal growth at the growth plate. The new bone formation occurs by two processes. New cortical bone is deposited around the periphery of the bone by osteoblasts, which form within the periosteum, and secrete compact bone matrix, resulting in widening of bone dimensions. Longitudinal growth occurs via endochondral ossification, the replacement of cartilage of the epiphyseal disks with new bone. In this process, as older chondrocytes within the epiphyseal disk die, and osteoblasts enter and lay down new bone to replace the old cartilage. Meanwhile, chondrocytes continue to divide and generate new cartilage, and the endochondral ossification process continues until closure of the epiphyseal plate occurs at the conclusion of growth. Once peak bone mass has been reached in early adulthood, bone remodeling becomes dominant and continues throughout life. Bone remodeling is a cyclic process of bone resorption and formation during which osteoclasts break down old bone and osteoblasts replace it with new bone at the same site, repairing the microarchitecture of existing bone, without net accrual of bone mass (4).

As kidney function declines, alterations in mineral and endocrine homeostasis occur, leading to abnormalities in the bone modeling and remodeling processes. Traditionally, evaluation of bone disease in CKD was limited to classification according to rates of bone turnover (increased bone turn over, decreased bone turnover or adynamic bone disease, and mixed). However, the importance of also assessing alterations in skeletal mineralization and bone volume were recently recognized. Disturbed mineralization and/or bone volume can be present in children with CKD, and associated complications including bone pain, deformities, and fractures may occur even in the setting of normal bone turnover (5–7). As such, kidney disease improving global outcome (KDIGO) recommends that the term “CKD-MBD” be used to describe the broad clinical syndrome encompassing mineral, bone, and calcific cardiovascular abnormalities that develop as a complication of CKD, and the term “renal osteodystrophy” is restricted to describe bone pathology associated with CKD. The evaluation and diagnosis of renal osteodystrophy requires bone biopsy, using a classification system based on parameters of bone turnover, mineralization, and volume (TMV) (4).

While bone biopsy remains the gold standard for assessment of bone quality, it is rarely performed in clinical practice due to the invasive nature of the procedure. Current options for non-invasive bone imaging include dual X-ray absorptiometry (DXA) and peripheral quantitative computed tomography (pQCT). DXA is a two-dimensional technology that measures bone mineral content (BMC) and bone area (BA), and then calculates areal BMD. DXA has numerous limitations, most notably the inability to distinguish between cortical and trabecular bone, and the underestimation of BMD in shorter individuals. Studies measuring BMD by DXA in pediatric renal transplant recipients have used height-adjusted BMD *Z*-scores to compensate for this limitation (3, 8, 9). pQCT uses quantitative computed tomography technology to measure volumetric BMD of peripheral bones. Advantages of pQCT over DXA technology include the ability to measure true volumetric bone density, and the ability to differentiate between cortical and trabecular bone, allowing for geometric measurements of total cross-sectional area of cortical bone and cortical thickness. The major disadvantage of pQCT is that it is more costly than DXA. Radiation exposure from both DXA (0.004–0.005 mSv) and pQCT (0.01 mSv) is low, however, pQCT radiation exposure is higher when compared with DXA (10).

Biochemical markers are another available tool for non-invasive evaluation of bone health. Traditionally, guidelines for children with CKD recommend routine monitoring of serum levels of calcium, phosphorus, alkaline phosphatase, and parathyroid hormone (PTH) before and after kidney transplant, but their value as biomarkers has been limited to the assessment of bone turnover (11, 12). A recent study used classification and regression tree analysis to establish biochemical markers as predictors of different histologic TMV lesions of renal osteodystrophy in pediatric dialysis patients (7). They reported that PTH and alkaline phosphatase levels <400 IU/L best predicted normal bone turnover and normal bone mineralization in this population. Higher PTH levels and lower calcium levels were associated with defective mineralization, irrespective of bone turnover (7). Another study showed that hyperparathyroidism was associated with decline in cortical volumetric BMD in children with CKD which was associated with a fourfold increased fracture risk (13).

Further studies are needed to identify new biomarkers and establish more precise target ranges for combinations of known biomarkers that can be used as non-invasive tools for the evaluation of TMV abnormalities in children with CKD.

## CKD-Related Bone and Mineral Changes

Alterations in bone and mineral metabolism begin early in the course of CKD. Bone histomorphometry shows defective skeletal mineralization is present in 29% of children as early as CKD stage 2, and progresses to 42% at stage 3, and 79% in stage 4 and 5 (14). Among pediatric dialysis patients, histomorphometry indicates increased bone turnover in 57% and abnormal bone mineralization in 48% of the population (7).

Traditional risk factors for CKD–BMD include vitamin D deficiency, secondary hyperparathyroidism, disturbance of the growth hormone (GH)/insulin-like growth factor-1 (IGF-1) axis, delayed puberty, malnutrition, and metabolic acidosis. Newer evidence indicates that fibroblast growth factor 23 (FGF-23) also plays a key role, and emerging knowledge suggests a role of the colonic microbiome in bone health.

### Vitamin D, calcium, phosphorus, and PTH

Adequate intake of 25(OH)D_3_ (nutritional vitamin D) and its final conversion to active 1,25-(OH)_2_D_3_ by 1-alpha-hydroxylase in the kidney, and calcium, phosphorus, and PTH play an important role in bone health. In children with CKD, the homeostatic mechanisms that regulate these are disrupted, leading to development of secondary hyperparathyroidism and renal osteodystrophy (4).

In the early stages of CKD, production of 1,25-(OH)_2_D_3_ may be impaired by elevated serum phosphorus and loss of 1-alpha-hydroxylase activity, in conjunction with inhibition of production and increased degradation of 1,25-(OH)_2_D_3_ by FGF-23 (15). Low 1,25-(OH)_2_D_3_ decreases serum calcium levels, leading to secondary hyperparathyroidism.

In addition, deficiency of nutritional vitamin D contributes to decreased production of 1,25-(OH)_2_D_3_ and is also directly associated with more severe secondary hyperparathyroidism along the entire spectrum of CKD, both before and after kidney transplant. Even when there is little or no residual 1-alpha-hydroxylase activity in the late stages of CKD, nutritional vitamin D deficiency leads to more marked secondary hyperparathyroidism (12, 16).

Dietary phosphorus load is another important determinant of severity of hyperparathyroidism, beginning in the early CKD stages, as high dietary phosphorus intake worsens hyperparathyroidism, even in the setting of normal serum phosphorus (12). Conversely, low serum phosphorus levels are also harmful, as adequate phosphorus balance is required for optimal bone mineralization and growth.

The role of PTH as a biomarker of renal osteodystrophy is an area of intense debate and ongoing research, and its diagnostic value is unclear. Based on the available evidence, KDIGO guidelines recommend maintaining PTH levels with the range of approximately 2–9 times the upper limit of normal. While the optimal target PTH value in CKD is not exactly known, markedly high or low values can be useful in predicting bone turnover (4). Further research is needed to establish more precise target ranges, and to clarify associations with TMV parameters, and outcomes, such as fracture.

### Metabolic acidosis

Metabolic acidosis has numerous negative effects on bone health. It can occur in the early stages of CKD due to underlying tubular disorders and obstructive uropathies. Additionally, in the late stages of CKD, as kidney function declines, the ability of the kidney to excrete ammonia is reduced, resulting in metabolic acidosis. The decrease in systemic pH induces calcium efflux from bones and increase in urinary calcium excretion, which serves to buffer hydrogen ions at the expense of bone mineral stores. Chronic metabolic acidosis inhibits osteoblastic and increases osteoclastic activity, impairs the incorporation of hydroxyapatite in bone, and causes resistance to both endogenous and recombinant human growth hormone (rhGH) (17). Chronic metabolic acidosis also alters the homeostatic relationships between calcium, PTH, and 1,25-(OH)_2_D_3_, promoting excessive bone dissolution (18). Consequences of chronic metabolic acidosis include bone fractures, reduction in BMD and bone formation rates, and reduced linear growth (19). Treatment of acidosis with bicarbonate has been shown to improve hyperparathyroidism and metabolic bone disease in CKD (20, 21).

### GH/IGF-1 axis

In children with CKD, disturbances in the GH/IGF-1 axis interfere with normal bone metabolism and growth. The pathogenesis involves GH insensitivity, evidenced by inappropriately high GH levels in the setting of diminished longitudinal growth, and decreased bioavailability of IGF-1 in patients with CKD. As kidney function declines in CKD, decreased renal clearance and increased hepatic production of IGF binding protein occurs. The accumulation of these high-affinity IGF binding proteins bind and inactivate free IGF-1, resulting in a functional deficiency of bioactive IGF-1, thereby inhibiting the action of IGF-1 on growth plate chondrocytes resulting in impairment of growth (22).

### Delayed sexual maturation and malnutrition

Sexual maturation is a key determinant of bone mass and strength, and adequate levels of sex hormones are required for growth and achievement of peak BMD. Estrogen is essential for accelerating longitudinal bone growth at the beginning of puberty and for ossification of cartilage into bone, leading to closure of the epiphyseal plate at the conclusion of growth in both males and females. Estrogen also promotes apoptosis of osteoclasts and inhibits apoptosis of osteoblasts to inhibit bone breakdown throughout the lifespan (23). In males, estrogen is produced from testosterone, via the enzyme aromatase. Testosterone is important for skeletal growth because of its direct anabolic effects on bone, as well as its ability to stimulate muscle growth, which puts greater stress on bone to promote increased bone formation (24).

In children with CKD, testosterone and estrogen production are impaired, predisposing them to delayed sexual maturation. The onset of puberty and the pubertal growth spurt in adolescents with CKD are delayed by 2.5 years on average (25). Studies have shown that pubertal timing predicts both cortical and trabecular volumetric BMD in young men, and late puberty is a risk factor for low BMD and fractures (26). In young women, delayed menarche is associated with lower bone density at the lumbar spine and femoral neck, and declines further with each year that menarche is delayed (27). A study of pediatric ESRD patients showed that age at menarche was significantly positively correlated with uremia duration (28). In boys with CKD and delayed puberty, treatment with exogenous testosterone resulted in development of sex characteristics and accelerated bone age that exceeded growth velocity, resulting in lost growth potential (29).

In concert with hypogonadism, malnutrition is also a common feature of children with CKD that contributes to delayed sexual maturation and impaired bone development and mineralization during growth. Poor nutrition and low body fat, or an altered ratio of lean mass to body fat, delay the adolescent growth spurt and retard the onset of menarche, as the initiation of menarche and maintenance of regular menstrual cycles are dependent on conditions of adequate body fat (30).

Nutritional deficiencies may also directly impact bone mineralization and growth. Children with CKD are at risk for macro and micronutrient deficiencies due to a multitude of factors including dietary restrictions, appetite suppression related to uremic inflammation and alterations in endocrine and neuropeptide signaling, and removal of protein, vitamins, and trace elements by dialysis (31). Inadequate dietary intake of calcium and phosphorus results in the formation of weak, poorly mineralized bone, and nutritional vitamin D deficiency leads to rickets and osteomalacia (24). Inadequate dietary intake and low serum levels of the essential trace elements zinc and copper have been reported in pediatric CKD and dialysis patients (32, 33). Zinc, which plays an important role in growth and sexual function, is depleted with dialysis, and up to 78% of dialysis patients are deficient (34). Zinc deficiency adversely affects bone metabolism and gonadal function, resulting in growth retardation and hypogonadism (35). Copper plays an important role in collagen cross-linking and bone formation, and copper deficiency is associated with metaphyseal abnormalities, osteoporosis, and fractures in children (36).

#### Fibroblast growth factor 23

Newer evidence indicates that FGF-23 plays a key role in the pathogenesis of renal bone disease during CKD. During early stages of CKD, alteration in mineral metabolism is marked by increasing FGF-23 and decreasing levels of co-receptor Klotho, which act in concert to induce renal phosphate excretion, suppress renal 1-alpha-hydroxylase activity, and inhibit PTH secretion in attempt to maintain phosphorus homeostasis (37–40). As GFR declines, FGF-23 loses ability to counteract phosphate retention, but FGF-23 levels continue to rise, reaching elevations of 100- to 1000-fold above normal in dialysis patients (41). This scenario predisposes the patient to tertiary FGF-23 excess and altered mineral metabolism characterized by post-transplant hypophosphatemia.

A short-term trial studied the effect of early administration of phosphate binders (sevelamer hydrochloride or calcium acetate) to normophosphatemic adults with stage 3 and 4 CKD. Results showed reduction in PTH levels without change in phosphorus or calcium levels in all patients, and significant decrease in FGF-23 levels in the group treated with sevelamer hydrochloride. Long-term studies are needed to determine whether early administration of phosphate binders to pediatric CKD patients with normal phosphate levels may provide long-term benefits to bone health, by targeting FGF-23 early in the course of CKD (42). Calcimimetics are useful in adults in controlling hyperparathyroidism but are not currently approved for use in children, as safety and long-term effects on bone have yet to be established.

#### Microbiome

The healthy intestine contains an ecosystem of microorganisms, comprised of more than 500 different species of bacteria, the delicate balance of which affects numerous organs and systems throughout the body. New research on the horizon involves study of the relationship of CKD and the colonic microbiome, and emerging evidence suggests that alterations in the microbiome occur that may contribute to derangement of bone and mineral metabolism. In the colon, anaerobic bacteria ferment undigested protein to produce a variety of potentially toxic metabolites, including indoles; these metabolites accumulate as renal function deteriorates. Indoles are absorbed and further metabolized to indoxyl sulfate in the liver (43). Indoxyl sulfate was shown to induce skeletal resistance to PTH by inducing osteoblast dysfunction *in vitro*, and prevention of accumulation of indoxyl sulfate improved bone formation in animal studies (44, 45). Further studies are needed to investigate the implications of microbiome alterations in CKD and potential future therapeutic targets in this area.

## Post-Transplant

Many children come to transplant with deranged bone health related to CKD. After transplant, recovery of bone health may be impaired by nutritional vitamin D deficiency, persistent hyperparathyroidism, tertiary FGF-23 excess, hypophosphatemia, immunosuppressive therapy with corticosteroids and/or calcineurin inhibitors (CNI), and alteration of sex hormones. The incidence of vertebral fracture in children after kidney transplantation is 140 times higher than the general population (46). Bone histomorphometry of pediatric kidney transplant recipients showed that defective skeletal mineralization was present in 34% (47). Recent studies measuring BMD by DXA have demonstrated that low BMD is common in pediatric renal transplant recipients (3, 8, 9). A recent study used pQCT to examine changes in trabecular and cortical volumetric BMD and cortical dimensions in children and adolescent renal transplant recipients for 12 months post-transplant, and found persistent deficits in cortical dimensions, and reduced trabecular BMD in the older recipients (2).

### Nutritional vitamin D deficiency

The prevalence of nutritional vitamin D deficiency among pediatric transplant recipients is high and more common compared to healthy controls (16). Recent studies report deficiency in 22–38% and insufficiency in 32–54% of transplanted children (3, 16, 48, 49). The role of vitamin D in bone mineralization and growth during childhood and adolescence is well established. In the pediatric renal transplant population, vitamin D deficiency has also been shown to correlate with hyperparathyroidism, short stature, and hypophosphatemia (48, 49). Studies show that nutritional vitamin D supplementation improves BMD in adults in both the general and renal transplant populations (50–52).

### Increased PTH, FGF-23, and hypophosphatemia

The phenomenon of early post-transplant hypophosphatemia related to decreased phosphorus reabsorption in the proximal tubule is well known, and persistence of hypophosphatemia in long-term kidney transplant recipients has been recently characterized (53–55). Excess of both FGF-23 and PTH have been found to contribute to post-transplant hypophosphatemia. However, there is controversy about whether PTH or FGF-23 plays a dominant role in post-transplant hypophosphatemia. In the early post-transplant period, Evenepoel et al. demonstrated that elevated pre-transplant FGF-23 levels were the strongest predictor of post-transplant elevation of FGF-23, and FGF-23 independently predicted hypophosphatemia and inappropriately low 1,25-(OH)_2_D_3_ levels during the first 3 months post-transplant (56). Further studies are needed to elucidate the pathogenesis of persistent long-term post-transplant hypophosphatemia, as some studies have reported that persistent hyperparathyroidism has a greater influence than FGF-23, while others suggest that FGF-23 is the main driving force behind the persistent dysregulation of phosphate homeostasis (53, 55, 57). In children with chronic allograft nephropathy, FGF-23 levels increase with the degree of chronic allograft failure, preceding increase in PTH, suggesting that FGF-23 may be a more sensitive biomarker of post-transplant phosphorus dysregulation and continuation or reactivation of CKD–MBD (55).

Persistent renal phosphate wasting in the setting of long-term post-transplant hypophosphatemia contributes to decreased osteoblast activity and progressive bone demineralization, increasing the risk of low BMD over time (58). More research is needed to determine the relationships between PTH, FGF-23, and hypophosphatemia with TMV bone parameters, and to identify possible therapeutic targets. There is also need for determining the optimal serum phosphorus level to maintain adequate bone mineralization after transplant.

### Hypomagnesemia

Hypomagnesemia is common after kidney transplantation, related to magnesium wasting secondary to use of CNI. Magnesium is an integral component of the hydroxyapatite structure of bone, and is essential for the physiological function of osteoblasts and osteoclasts and regulation of PTH. Magnesium deficiency contributes to osteoporosis by acting on crystal formation and bone cells impairing the magnesium dependent hydrogen-potassium-ATPase pump within the cells of the periosteum, leading to decreased pH of extracellular bone fluid and increased bone demineralization. Magnesium deficiency has numerous effects on PTH regulation, which include impairing PTH secretion, inducing PTH resistance and decreasing production of 1,25-(OH)_2_D_3_ (59). In addition, hypomagnesemia has been reported to cause hyperparathyroidism in some studies. The mechanism of action of magnesium in modulating excess PTH secretion mimics that of calcium at the calcium sensing receptor of the parathyroid glands. Indeed, a prospective long-term study of renal transplant recipients showed that hypomagnesemia at 5 years post-transplant is an independent predictor of persistent hyperparathyroidism (54). Low magnesium levels have been linked with decreased BMC and BMD in malnourished children (60), and magnesium supplementation resulted in increased accrual of bone mass in peripubertal girls with suboptimal dietary magnesium intake (61).

### Effect of immunosuppression

All maintenance immunosuppressive therapies have been implicated in causing impairment of bone health in children after transplant. Corticosteroids inhibit bone formation by decreasing gastrointestinal calcium absorption, reducing osteoblast proliferation, inducing osteoblast apoptosis, impairing osteoblast function via interference with the GH/IGF-1 axis, and promoting osteoclastogenesis; these changes result in decreased bone formation, bone loss, and increased risk for fractures (62). A study of changes in BMD after renal transplant in children reported decreased trabecular and increased cortical BMD associated with greater glucocorticoid exposure, reflecting the preferential depletion of trabecular bone volume fraction by glucocorticoids (2). A large case–control study of over 37,000 children treated with four or more courses of oral corticosteroids found that, compared to controls, corticosteroid-treated children had an adjusted odds ratio for fracture of 1.32 (63). The introduction of steroid avoidance protocols in the early 2000s has resulted in a decrease in the use of post-transplant steroid immunosuppression therapy by almost 20%, according to an analysis of the North American Pediatric Renal Trials and Collaborative data over 20 years. In this analysis of over 10,000 pediatric transplant recipients, growth improved with steroid avoidance, height *Z*-score remained stable with alternate day steroid therapy, and declined with the daily use of steroids (64).

CNI medications, such as tacrolimus and cyclosporine, also exert negative effects on bone by inhibiting synthesis of the vitamin D receptor and osteoprotegerin and stimulating osteoclast differentiation (65). Tacrolimus and cyclosporine have been linked to osteoporosis in adult renal transplant recipients (66). In addition, CNI have been associated with post-transplant bone pain syndrome, possibly due to intraosseous vasoconstriction and ischemia (67). Evidence from *in vitro* and animal studies indicate that mammalian target of Rapamycin (mTOR) inhibitors including sirolimus and everolimus may also impair bone formation and growth by interfering with osteoblast proliferation and inhibiting growth plate structure and function (68, 69). Rats treated with sirolimus showed signs of decreased bone longitudinal growth rate, reduced chondrocyte proliferation, disturbed chondrocyte maturation, decreased cartilage resorption, and morphological evidence of altered vascular invasion in the growth plate (69). However, in a recent clinical trial, longitudinal growth over 2 years in steroid-free pediatric patients on low-dose everolimus and cyclosporine was not different to that of a matched steroid-free control group on an immunosuppressive regimen with standard-dose CNI and mycophenolate mofetil. Further studies are needed to elucidate the effect of mTOR inhibitors on bone (70).

### Role of sex hormones

Delayed sexual maturation may remain a problem after kidney transplantation, impeding improvements in bone development and mineralization post-transplant. A study assessed sexual maturation of pediatric kidney transplant recipients by Tanner staging and found that the age of onset of puberty was significantly delayed in the transplant recipients compared with controls (28). Delayed sexual maturation was present in 22.2% of girls and 19.1% of boys, and was related to lower levels of sex steroids in these transplant recipients. Delayed post-transplant sexual maturation may be attributed in part to glucocorticoid therapy, which is known to reduce the production of sex hormones and interfere with bone maturation by impairing differentiation of the growth plate (71). Thus, in patients receiving steroid therapy after kidney transplantation, improvements in bone maturation and growth may not occur (25).

### Role of microbiome

After transplant, a variety of factors may continue to impact the gastrointestinal microbiota. Prophylactic antibiotic therapy induces modification of gut flora that results in reduction in mineral solubility, absorption, and decreased mineral deposition in the skeleton (72). Early evidence suggests that treatment with prebiotics and probiotics may counteract antibiotic-induced effects by restoring the colonic microbiota and cecal pH, stimulating mineral solubility and absorption, and promoting improvement in BMC and structure in both animals and humans (72). Probiotics, including Bifidobacteria and Lactobacilli, have been accepted as generally safe for use in healthy infants and children, but more data are needed to establish safety for individual probiotic strains in immunocompromised populations, such as pediatric transplant recipients (73). Cases of bacterial sepsis related to probiotic use, including Lactobacillus and Bifidobacterium strains, have been reported in infants and children with underlying immune compromise or chronic disease, and therefore use in these patients merits further study to establish safety and efficacy in the pediatric transplant population (74, 75).

## Current and Future Strategies for Optimization of Post-Transplant Bone Health

The optimization of bone health after transplantation can only be achieved through regular monitoring and careful management of the myriad of components that contribute to CKD–BMD, beginning in the early stages of CKD, and continuing throughout ESRD, at the time of renal transplant, and beyond.

### Evaluation and monitoring

Measurement of BMD in the first 3 months after kidney transplant should be considered in patients who receive corticosteroids or have risk factors for osteoporosis as in the general population (76).

Regular monitoring of serum calcium, phosphorus, total CO_2_, alkaline phosphatase, PTH, and 25(OH)D should begin at stage 2 CKD and continue throughout the cycle of CKD, dialysis, and transplantation (11). In the immediate post-transplant period, it is recommended to monitor serum calcium and phosphorus levels at least weekly, and thereafter, monitor these and other parameters, including magnesium, based on the degree of abnormalities and rate of progression of CKD (76).

### Promotion of healthy diet and lifestyle

Adequate dietary calcium intake during childhood and adolescence is recommended for skeletal development and acquisition of bone mass; however excessive calcium should be avoided, as positive calcium balance contributes to development of soft-tissue calcifications. KDOQI guidelines advise adequate dietary intake of calcium, phosphorus, and vitamin D to meet at least 100% of the DRI for age, and not to exceed 200% of the DRI for age for calcium (Table 1) (12). The recommended daily intake for vitamin D is 400 international units for infants and 600 international units for ages 1–30 years of age. It is recommended that exclusively breast fed infants as well as those taking <1000 mL/day of fortified formula should be supplemented with 400 international units of vitamin D daily (77).

**Table 1:** **Dietary reference intakes (DRI) for calcium and phosphorus (77)**.

Age	Calcium DRI (mg/day)	Phosphorus DRI (mg/day)	Copper DRI (mcg/day)	Zinc DRI (mg/day) female/male	Magnesium DRI (mg/day) female/male
0–6 months	200	100	200	2	30
6–12 months	260	275	220	3	75
1–3 years	700	460	340	3	80
4–8 years	1000	500	440	5	130
9–13 years	1300	1250	700	8	240
14–18 years	1300	1250	890	9 (F)/11 (M)	360 (F)/410 (M)

Guidelines currently recommend a high phosphorus diet and oral phosphate supplementation for children with persistent post-transplant hypophosphatemia. However, more recent data suggest that oral phosphate supplementation may be ineffective in correcting post-transplant hypophosphatemia, as it further stimulates FGF-23 secretion. Research and development of novel therapies are needed. Anti FGF-23 antibodies have been shown to lower FGF-23 levels and improve hypophosphatemia in animal studies and may be an area of further research to improve bone mineralization after transplant (78). In another animal study, an FGF-23 receptor inhibitor was used to block the FGF-23 receptor, resulting in significantly lower FGF-23 levels compared with control animals, representing another possible therapeutic option (79).

For promotion of bone health, adequate dietary intake to meet 100% of the DRI for minerals that affect bone health, including copper, zinc, and magnesium, is recommended (Table 1) (12). Post-transplant hypomagnesemia should be corrected with oral magnesium supplementation to achieve and maintain magnesium homeostasis and promote optimization of BMD (12, 59).

Following a low sodium diet has been shown to promote beneficial skeletal effects, while high sodium intake increases urinary calcium excretion and loss of calcium from bone, decreases bone formation, and is associated with reductions in plasma calcium and 1,25-(OH)_2_D_3_ (80). Because BMD is inversely related to sodium intake, and additionally as hypertension is common after transplant, pediatric renal transplant recipients may benefit from limiting sodium intake to no more than 2000 mg/day. Interestingly, omega-3-fatty acids were shown to counteract the bone alterations induced by high salt intake, evidenced by increased bone mass and formation and decreased bone resorption and bone loss in omega-3 fatty acid supplemented rats on a high salt diet, compared with rats on high salt diet alone (80). These beneficial effects of omega-3 may be attributed to its positive effects on calcium, phosphorus, and alkaline phosphatase homeostasis, and could provide a non-toxic therapeutic option if proven effective in human studies.

Avoidance of cola beverages is also recommended. The intake of cola beverages has been linked with decreased BMD and increased fracture risk in the pediatric population, and with increased risk of osteoporosis in adult women. In animal studies, cola-fed rats displayed decreased osteogenesis, delayed bone formation, and thinner trabecule compared with controls (81). Possible contributors to the deleterious effects of excess cola consumption on bone may include the replacement of more nutrient-rich foods and beverages in the diet by cola, reduction of vitamin D synthesis and calcium absorption by the phosphoric acid in the cola, and/or accelerated bone resorption induced by the acid load of the cola. The fructose in cola may also compromise bone health in growing children, based on data demonstrating decreased levels of 1,25-(OH)_2_D_3_, decreased bone length and bone ash, and increased FGF-23 levels in growing fructose-fed rats. In this study, the decreased 1,25-(OH)_2_D_3_ levels were associated with reduction in calcium transport rate and expression of intestinal and renal calcium transporters (82).

Weight-bearing exercise plays a key role in maintaining bone mass throughout life, and is important for promoting bone health and strength after transplant. Bones strengthen in response to mechanical loading forces, but weaken if not subjected to loading and weight bearing for sufficient periods of time. Moderate weight-bearing physical activity of a minimum of 30 min on most days of the week, to include short bouts of high-impact activity such as skipping or jumping, is recommended to promote increase or preservation of bone mass (24).

### Correction of vitamin D deficiency and PTH abnormalities

Nutritional vitamin D deficiency/insufficiency should be treated with oral supplementation, both before and after renal transplantation, however the optimal treatment regimen is not known. Guidelines for the pediatric CKD population have been established (Table 2) (12). There are presently no specific guidelines for the pediatric transplant population. The level of vitamin D sufficiency is not well defined, with the target levels varying from >20 to 30 ng/mL (4, 12, 83). There is some evidence to suggest that cholecalciferol is more effective than ergocalciferol for repletion of vitamin D (3, 84).

**Table 2:** **Treatment for 25-hydroxy vitamin D insufficiency/deficiency in CKD (12)**.

25-Hydroxy vitamin D level (ng/mL)	Cholecalciferol treatment dose
<5	8000 IU/day × 4 weeks, then 4000 IU/day × 8 weeks
5–15	4000 IU/day × 12 weeks
16–30	2000 IU/day × 12 weeks

Parathyroid hormone levels should be maintained within the target range based on stage of CKD (Table 3). As allograft function declines, 1,25-(OH)_2_D_3_ should be initiated if nutritional vitamin D is replete and PTH is above the target range for CKD stage (11).

**Table 3:** **Target range of PTH for stage of CKD (11)**.

CKD stage	Target serum PTH (pg/mL)
2–3	35–70
4	70–110
5	200–300

#### Correction of metabolic acidosis

Metabolic acidosis after kidney transplantation may occur due to CNI effects and acute rejection, and contributes to post-transplant abnormalities in calcium and phosphorus metabolism and disorders of bone remodeling via skeletal buffering of excess protons. Acidosis should be corrected and CO_2_ level maintained ≥22 mEq/L to promote resolution of electrolyte abnormalities, decrease the risk of post-transplant osteoporosis, and maximize growth (19).

#### Recombinant human growth hormone therapy

Recombinant human growth hormone (rhGH) therapy has been established as an effective therapy for improvement of growth velocity in children with CKD, including those post-transplant (85, 86). More recent studies have investigated the effects of rhGH on bone mineralization. A study of infants with CKD found that those treated with rhGH for 12 months had improved BMD, evidenced by a 39% increase of radius mineral density compared with baseline (87). Another study evaluated BMD distribution in children and adolescents with ESRD on dialysis using quantitative backscattered electron imaging of paired bone biopsy specimens and found that 1 year of therapy with rhGH resulted in improved growth and histomorphometric indices of bone formation rate, suggesting improved bone matrix mineralization (88). Data on the relationship between rhGH and BMD in the pediatric transplant population are scarce. One study of pediatric renal transplant recipients found that treatment with rhGH maintained vertebral bone mass, whereas modest decrease in bone mass were observed in untreated control subjects (89). Recent analysis of a large retrospective pediatric cohort indicated that rhGH is not associated with increased risk of post-transplant lymphoproliferative disorder (90), however, some data suggest rhGH may increase the risk of acute rejection, thus further investigation of safety of rhGH in the pediatric transplant population may be warranted (86).

#### Steroid avoidance

Kidney disease improving global outcome guidelines recommend minimizing or avoiding corticosteroid use in children who still have growth potential (76). A recent analysis of the United States Renal Data System data (*n* = 77430) reported that adult kidney transplant recipients on early steroid withdrawal protocols (discharged without corticosteroids) had a 31% lower fracture risk compared to those discharged with corticosteroid therapy (62). Prospective randomized clinical trials in pediatric kidney transplant recipients have shown that steroid avoidance protocols are safe and are associated with improved in growth in pre-pubertal recipients followed for up to 36 months post-transplant (91, 92). However, one prospective pediatric study showed that steroid minimization alone was not effective in preventing post-transplant bone mineral loss (93). The continued development of strategies to minimize steroid exposure in the pediatric transplant population is important.

#### Drug therapy

The KDIGO guidelines recommend consideration of vitamin D analogs or bisphosphonates to treat bone disease during the first year post-transplant in adults (4). Very little data is available on the safety and efficacy of treatment of post-transplant bone disease with vitamin D analogs and bisphosphonates in children. In a 2-year long randomized double-blind controlled trial of 34 children with osteogenesis imperfecta, bisphosphonate treatment was associated with a 31% reduction in relative risk of fracture of long bones, greater increase in spinal BMC and BMD compared with placebo (94). In the pediatric transplant population, a study of 30 pediatric transplant recipients with low BMD showed improved lumbar spine BMD after treatment with a 1,25-(OH)_2_D_3_ analog compared with placebo (95). In another study, pediatric transplant recipients treated with either alfacalcidol, alendronate, or calcitonin for 1 year demonstrated consistent improvement in BMD with all therapies compared to an untreated control group (96). Grenda et al. compared effect of three different interventions [prophylactic oral ibandronate with steroids, daily 1,25-(OH)_2_D_3_ supplementation with steroids, or steroid minimization] on the long-term BMC and BMD of pediatric renal transplant recipients. In this study, the patients who received the prophylactic ibandronate maintained BMC and BMD over the 2-year period, while those receiving 1,25-(OH)_2_D_3_ or steroid minimization demonstrated significant decrease in BMD (93).

In adult transplant recipients, testosterone and estrogen replacement have been shown to slow bone loss (67). KDOQI guidelines for adult transplant recipients suggest that therapy with replacement of gonadal steroid hormones for estrogen-deficient and testosterone-deficient patients should be seriously considered after kidney transplantation (19).

Teriparatide, a recombinant human PTH, is a potential treatment option in adult transplant recipients with low PTH and refractory hypocalcemia (97). Denosumab, a RANK-ligand inhibitor, inhibits the maturation of osteoclasts to protect bone from degradation, and is approved for treatment of osteoporosis in post-menopausal women, and can be used in post-menopausal women with stages 1–3 of CKD (98).

Presently, there is insufficient evidence to guide use of these drug therapies to treat bone disease in pediatric kidney transplant recipients.

#### Microbiome

Strategies to reestablish symbiosis of the colonic microbiome are under investigation and may hold promise as a new frontier in the development of future therapeutic interventions. Aims of potential therapies that may promote improvement in bone health include restoration of the bacterial milieu of the colon, reduction of generation of bacterial toxins in the colon, or adsorption of the toxic end products of microbial fermentation within the colon. In the general pediatric population, supplementation of infant formula with the probiotic Bifidobacteria reduced stool pH and decreased fecal ammonia and indoles in healthy infants (99, 100). In the adult CKD and dialysis populations, prebiotic and probiotic therapies were shown to reduce serum concentrations of the toxin indoxyl sulfate, thereby promoting improved bone formation (43, 101). A vegetarian diet was also recently associated with lower production of indoxyl sulfate in healthy adults (102). Adsorption therapy using AST-120, an oral carbon-based adsorbent material, was shown to reduce serum indoxyl sulfate levels in animals, as well as in adult CKD patients, but requires further study (103, 104). There are no published studies investigating the effects of these therapies in pediatric CKD or dialysis populations, and therapies targeting the colonic microbiota of the pediatric transplant population also remain an unexplored area.

## Conclusion

The accrual of optimal bone mass during childhood and adolescence is the best protection against development of osteoporosis later in life (1), however achieving this in children with CKD is challenging. Loss of kidney function engenders a chain of disturbances in endocrine and mineral metabolism that require precise monitoring and management in order to preserve bone health. After transplantation, new factors, including effects of immunosuppressive therapies pose continued threats to bone. Little is known about effective therapies for treatment of bone disease in the pediatric CKD and transplant patients, therefore continued efforts toward research and discovery in this field is critical for improving bone health of this population.

## Key Points

- Disturbances in mineral metabolism and endocrine homeostasis begin early in the course of CKD, leading to CKD–BMD, and impaired growth in children.
- Risk factors for CKD–MBD include nutritional vitamin D deficiency, secondary hyperparathyroidism, increased FGF-23, altered GH/IGF-1 axis, delayed puberty, malnutrition, and metabolic acidosis.
- After kidney transplantation, nutritional vitamin D deficiency, persistent hyperparathyroidism, tertiary FGF-23 excess, hypophosphatemia, hypomagnesemia, immunosuppressive therapy, and alteration of sex hormones continues to impair bone health and growth.
- Bone biopsy is the gold standard for evaluation of bone quality, which should be defined by assessment of bone turnover, mineralization, and volumetric parameters. DXA and pQCT are non-invasive options, but carry limitations.
- Optimal target ranges for combinations of traditional biomarkers (serum calcium, phosphorus, PTH, and alkaline phosphatase), and newer biomarkers, such as FGF-23, to predict bone quality need to be investigated.
- Strategies to optimize bone health post-transplant include healthy diet, weight-bearing exercise, correction of vitamin D deficiency, acidosis, and electrolyte abnormalities, steroid avoidance, and consideration of rhGH therapy when indicated.
- Drug therapies including vitamin D analogs, bisphosphonates, hormone replacement, teriparatide, denosumab, and calcimimetics have been used in adult transplant recipients, but there is insufficient evidence for use in the pediatric population at the present time.
- Future therapies to be explored include anti-FGF-23 antibodies, FGF-23 receptor blockers, and treatments targeting the colonic microbiota by reduction of generation of bacterial toxins or adsorption of toxic end products that affect bone mineralization.

## Conflict of Interest Statement

The authors declare that the research was conducted in the absence of any commercial or financial relationships that could be construed as a potential conflict of interest.
